# Human Microglial Molecular Alterations in Aging and Alzheimer’s Disease

**DOI:** 10.3390/cells15131159

**Published:** 2026-06-25

**Authors:** Karol Ann T. Baldo, Elisa Gozlan, Emmanuel O. Chidebe, Ifeoluwa Awogbindin, Marina Ocaña Prieto, Leslie Michelle Dalmacio, Benneth Ben-Azu, Dan Frenkel, Marie-Ève Tremblay

**Affiliations:** 1 School of Medical Sciences, Faculty of Health, University of Victoria, Victoria, BC V8P 5C2, Canada; ktbaldo1@up.edu.ph (K.A.T.B.);; 2 Department of Biochemistry and Molecular Biology, College of Medicine, University of the Philippines Manila, Manila 1004, Philippines; 3 Department of Neurobiology, School of Neurobiology, Biochemistry and Biophysics, George S. Wise Faculty of Life Sciences, Tel Aviv University, Tel Aviv 6997801, Israel; 4 DELSU Joint Canada-Israel Neuroscience and Biopsychiatry Laboratory, Department of Pharmacology, Faculty of Allied Health Sciences, Delta State University (DELSU), Abraka 330106, Delta, Nigeria; 5 Institute on Aging and Lifelong Health (IALH), University of Victoria, Victoria, BC V8P 5C2, Canada; 6 Centre for Advanced Materials and Related Technology (CAMTEC), University of Victoria, Victoria, BC V8P 5C2, Canada; 7 Groningen Institute for Evolutionary Life Sciences, University of Groningen, 9712 CP Groningen, The Netherlands; 8 Sagol School of Neuroscience, Tel Aviv University, Tel Aviv 6997801, Israel; 9 Department of Biochemistry and Molecular Biology, The University of British Columbia, Vancouver, BC V6T 1Z3, Canada

**Keywords:** microglia, human, aging, Alzheimer’s disease, oxidative stress, senescence, inflammation

## Abstract

**Highlights:**

- Human microglia in aged brain exhibit morphological and molecular perturbation that could be linked with a shift from homeostatic states to chronic reactivity, as well as dystrophy and impairment of physiological functions.
- Dysregulated microglia-mediated oxidative stress, pro-inflammatory states, and senescent milieu in aged human brain may pose a potential link between physiological brain aging and Alzheimer’s disease (AD)-related pathological trajectories.
- Microglial dysfunction in overall brain inflammation and AD pathogenesis underscores an imperative priority in the advancement of microglia-targeted therapeutic strategies to modulate pathology and disease progression.

**Abstract:**

Microglia, the resident innate immune cells of the central nervous system, are central players in brain development, healthy aging, and degenerative pathology, including Alzheimer’s disease (AD). Aging is a major risk factor for AD, and various studies have identified alterations in microglial molecular signatures and morphological patterns that overlap with microglial states during aging. However, the mechanisms underlying the divergence of aging trajectories toward disease remain unclear. Thus, understanding the molecular changes in microglia during aging and AD pathology is crucial to elucidating the mechanisms that drive disease progression. In this review, we examine current advances in understanding the phenotypic alterations in human microglia, highlighting gene signatures and morphological changes that may aid in defining microglia’s molecular and functional programs in healthy aging and over the course of AD. We further explore the roles of oxidative stress and cellular senescence in driving the development of a chronic reactive state in microglia during aging, which may also contribute to the complex process underlying the onset and progression of AD pathology. This review highlights the advancements in therapeutic strategies focused on targeting pertinent pathological microglial changes during aging and in disease to mitigate the AD neurodegenerative process.

## 1. Introduction

The proportion of the global elderly population is continuously increasing [1]. According to the World Health Organization (WHO), 1 in 6 individuals globally will be aged over 60 years by 2030 [2]. This age distribution is estimated to double from approximately 1 billion in 2020 to 2.1 billion by 2050, potentially owing to increases in longevity, notably considering the advancement in medicine, and decline in birth rates [2,3]. Aging is a progressive biological process characterized by a steady decline in physiological functions across various organ systems. However, it is accompanied by a rising prevalence of age-associated diseases, with extensive health, societal, and economic burden [1,4,5]. Alzheimer’s disease (AD), a neurodegenerative disorder associated with progressive cognitive decline, is one of the leading causes of morbidity and mortality among older adults worldwide; aging remains its most significant risk factor [6,7,8]. AD affects more than 55 million people globally and, therefore, remains an international public health concern [9]. Despite considerable progress in AD research, positive outcomes for disease-modifying therapies remain limited, underscoring a pressing demand to identify mechanisms that can slow or halt disease progression. It is thus imperative to thoroughly evaluate the mechanisms underlying biological aging to better understand the processes that could drive the shift toward age-related disease states like AD [8].

Recent advances increasingly implicate microglia, the resident macrophages of the central nervous system (CNS), as key contributors to both CNS aging and neurodegeneration [10]. Microglia are involved in brain development and lifelong plasticity through important beneficial functions, including CNS surveillance and homeostasis, innate immune defence, gliogenesis and neurogenesis, myelin turnover, synaptic maintenance and remodeling [11,12,13]. However, microglia exhibit age-associated changes revealed through altered molecular (e.g., transcriptional) and morphological states compromising their function [10]. Generally, these changes in age-associated cellular characteristics (e.g., mitochondrial and deoxyribonucleic acid (DNA) damage, telomere shortening, etc.) are consistent with cellular senescence, a state in which a cell undergoes permanent cell-cycle arrest while maintaining metabolic activity, which is implicated in microglia’s functional decline in the aged brain [14,15]. Impaired processing and clearance of neurotoxic proteins (e.g., amyloid-beta or Aβ) and an excessive immune response coinciding with chronic CNS inflammation, both linked to disease progression, are some of the microglial alterations similarly observed in aging and AD pathology [14,16]. Consistent with this, multiple AD risk loci identified by genome-wide association studies (GWAS) correspond to genes that are highly expressed in microglia [17]. Some of these genes were also found to be enriched in the aged brain in neurologically healthy individuals [17]. Nevertheless, the cellular and phenotypic changes that underlie microglial dysfunction during physiological aging and in age-related disease processes remain largely unelucidated [10,18].

With growing evidence that microglia adopt distinct age-associated states that may shape vulnerability to neurodegenerative diseases, defining how microglial properties change with aging, along with exposure to various noxious signals, is a critical research avenue. Microglial-mediated mechanisms have been more comprehensively characterized through studies utilizing mouse models of aging and neurodegenerative disease pathology. However, species differences between murine and human microglia in the context of aging have been recognized [19]. Whilst, a key gap remains in identifying microglial alterations that lead to cognitive decline in human aging and AD. Therefore, this review integrates perspectives from human studies and explores recent insights into the molecular and phenotypic landscape of human microglia during aging and examines how these changes relate to, or differ from, those occurring in AD. We further discuss the converging microglial molecular alterations linked with physiological and pathological processes in human aging, including oxidative stress, cellular senescence and inflammaging, to shed light on areas where current research disparities lie. All in all, we focus on current evidence describing aging-associated shifts in human microglial molecular, phenotypic, and functional profiles, with implications for identifying therapeutic strategies against cognitive impairment during aging and in AD.

## 2. The Aged Brain and Microglia

As the CNS ages, the brain undergoes progressive physiological changes that lead to cumulative alterations across neural networks [20], and this can contribute to gradual shifts in neurological functioning [21,22]. In this review, we refer to microglial characteristics as ‘aged’ or ‘aging’ broadly, based on phenotypic data from post-mortem human brains from individuals of advanced age, generally 60 years and older. Although a consensus is yet to be reached, a broad criterion is clinically used to distinguish physiologic from pathological aging, which the latter encompasses aging trajectories associated with marked functional decline in one or more cognitive areas associated with neurodegenerative processes like AD [20,23,24].

Age-related brain changes occur at multiple levels. For instance, aging at the molecular and cellular landscapes is associated with increased astrocytic and microglial activity, altered mitochondrial and intracellular calcium homeostasis, and oxidative stress, which can contribute to DNA and protein damage [25,26,27]. More broadly, these changes may contribute to a reduction of synaptic function and heightened immune activity, which appear to be prominent features of the aged human brain [28,29]. As the primary innate immune cell in the brain, maladaptive shifts to microglial core biological functions have been implicated in the development of these deleterious changes [10,21]. Moreover, because several of these features overlap with changes in AD, it remains critical to delineate where mechanisms diverge from, or converge with, physiologic aging processes. Hence, probing microglial molecular and cellular programs that could play a role in the process of brain aging or pathology may shed light on this unknown.

Microglia play essential roles in the maintenance of brain health, and thus are widely considered a vital mediator of CNS homeostasis, whilst their dysfunction has been linked to various disease states [30,31]. Microglia are derived from primitive macrophages that originate from the embryonic yolk sac, specifically erythromyeloid precursors, that migrate in a hematogenous manner to the neuroepithelium, where they expand and colonize the brain during embryogenesis [32,33]. Through local self-renewal, microglia are retained in the brain and spinal cord throughout adulthood and in advanced age [11]. In contrast, astrocytes and oligodendrocytes originate from the neuroectoderm (neural progenitors) and are replenished by differentiation from neural stem cells and oligodendrocyte progenitor cells (OPCs), respectively [34]. Accordingly, microglia are considered long-lived, tissue-resident innate immune cells in the CNS [35]. Consistent with this identity, age-associated shifts in microglial state are increasingly implicated in multiple CNS alterations during physiological aging and in AD pathology [36,37]. Importantly, the regional and temporal tissue microenvironment shapes microglial states (i.e., transcriptomic and morphologic) and their functional response [38]. Microglia located in specific neuroanatomical regions could be differentially vulnerable to these age-related changes and development of AD pathologies, such as the hippocampus and prefrontal cortex, which are important in memory and executive function, respectively, thereby leading to cognitive impairment [26,39]. Together, these direct our focus to microglial programs as a potential link between physiological brain aging and neurodegenerative disease [40,41].

Molecular and cellular features of aging cells, including chronic inflammation, cellular senescence, and oxidative stress, were previously referred to as “hallmarks of aging” [42]. In this review, we further highlight how some of these features may intersect with observed patterns of microglial dysfunction associated with aging and AD [10,16].

### 2.1. Microglial Morphological Alterations in Physiological Aging

In response to CNS microenvironmental cues, microglia dynamically shift between morphological states throughout life, which may correspond to distinct functions [10,38]. For instance, microglia generally display a ramified phenotype, characterized by numerous processes with pervasive branching patterns and small spherical cell bodies under homeostatic conditions [24]. Toxic proteins or noxious stimuli in the brain parenchyma may induce microglia to shift from surveilling states (dominantly displayed in a ramified morphology) toward an ameboid morphology (with rounded form, increased soma size, and retracted (if any) or absent processes) [38,43,44,45,46,47,48]. These morphologies can also transiently shift to other forms in a spatiotemporally regulated manner depending on the local tissue context and nature of the stimulus [38]. Although diverse microglial morphologies have been extensively characterized in animal models, particularly in mice, their dynamic features remain less comprehensively defined in humans [46].

**Figure 1:**
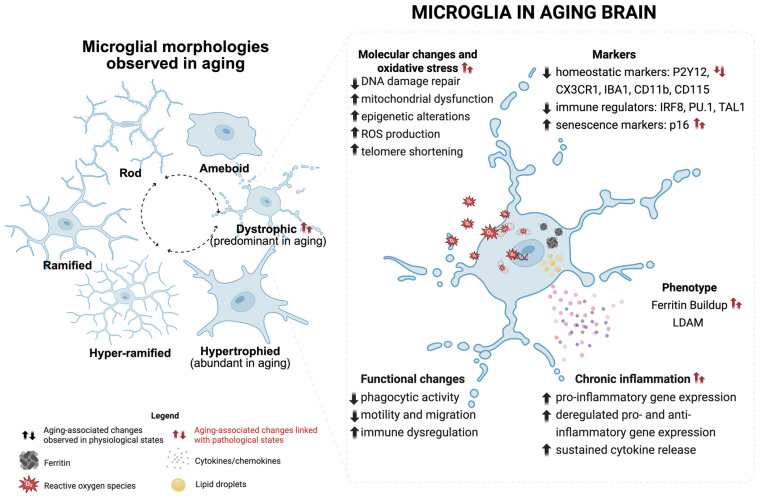
Microglial changes in physiological brain aging. Microglia in healthy aging in humans present hallmarks of cellular aging, notably exhibiting ferritin accumulation, sustained oxidative stress, and inflammatory states. Microglia remain diverse, with dystrophic and hypertrophic morphologies being the most abundant. Collectively, these microglial alterations in advanced age point to a shift from a homeostatic state to a chronically reactive state, accompanied by dysfunction, which could reinforce molecular processes that drive the cell to functional decline. Red arrows indicate microglial aging-related changes that could be linked with pathological states. Cell surface chemokine receptor 1 (CX3CR1); cluster of differentiation 68 (CD68); cyclin-dependent kinase inhibitor 2A/p16^INK4A^ (p16); interferon regulatory factor 8 (IRF8); ionized calcium-binding adapter molecule 1 (IBA1); lipid droplet accumulating microglia (LDAM); purinergic receptor P2Y, G-protein coupled, 12 (P2RY12); purine rich box binding protein 1 (PU.1); reactive oxygen species (ROS); T-cell acute lymphocytic leukemia protein 1 (TAL1). Created in BioRender.com (accessed on 16 June 2026).

Histological studies of post-mortem human brain indicate that microglial morphology changes with aging (Figure 1) [24,49]. Altered homeostasis, intercellular signaling, and the presence of disease-related processes can each influence the morphology adopted by microglia, which may reflect their physiological function during the chronological aging process. In advanced age, hypertrophic microglial morphology (thicker, shorter processes, less branching, smaller arborization area with enlarged soma) is frequently reported in both male and female post-mortem human neocortex (i.e., ages 52–98 years) [44,50,51], although multiple morphologies can coexist within aging within and across brain regions (Figure 1) [48]. Hypertrophic microglia have been associated with an increased expression of inflammatory markers and upregulation of genes related to cellular migration and phagocytosis [24,52,53,54]. On the other hand, ameboid microglia are also abundant in advanced age, while cell proliferation- and immune response-related genes are enriched in this form [4,38,48,52,55]. In response to injury, this state is often associated with heightened phagocytic activity and migratory capacity, accompanied by a robust secretory profile of inflammatory factors; therefore, it has been considered a “maximally reactive state” [48,52]. In some contexts, this morphology may transition from rod-shaped microglia, with elongated soma and polarized processes, associated with high phagocytic activity and proposed to play a role in synaptic stripping, which is the physical separation of pre- and post-synaptic compartments by intervening microglial processes [13,52,56]. It has been reported that advanced age is a strong determinant for the presence of rod-shaped microglia, particularly in the cerebral cortex and hippocampus, in post-mortem human brains [57]. They have also been detected in mouse models of disease, like amyotrophic lateral sclerosis and other neurodegenerative disease conditions [58]. In AD, while amyloid plaques and hyperphosphorylated tau influenced the presence of the rod-shaped microglia, correlation was only observed in specific cortical regions (i.e., parietal cortex) and, thus, they may not represent a distinct feature of AD-related pathology [57].

Another microglial morphological state that was observed to be increased in older adults is the dark microglia, showing an electron-dense cytoplasm and nucleoplasm, and a less discernible pattern of nuclear heterochromatin compared with typical microglia [59,60]. These cells were found to tightly associate with synapses and contain pre-synaptic components that could result in synaptic loss [59,60]. Observed in mouse models (i.e., male mouse hippocampus CA1 and frontal cortex) and in humans (both male and female frontal cortex), dark microglia have been characterized to exhibit a heightened prevalence of cellular stress markers, such as mitochondrial alterations, as well as dilation of Golgi apparatus and endoplasmic reticulum (ER) cisternae [59,60,61,62]. These cells notably display increased abundance following chronic psychological stress and in advanced age, as well as in AD pathology [59,60,61,62]. Considered a ‘transitional state’ between dark and typical microglia, intermediate microglia exhibit cellular stress features similarly to dark microglia but with less dense cytoplasm and nucleoplasm, and a more discernible heterochromatin pattern [62]. They have been posited to arise as an early cellular stress response in microglia [59,60,61,62], where exposure to chronic psychological stress or pathological input could lead to progression toward a dark microglial state. Together, these observations support a view of aging microglia as morphologically heterogeneous, with state transitions shaped by context-specific stimuli, including AD pathology [38,52].

Nevertheless, among other morphological forms, studies also report that microglia in the aged human brain are predominantly dystrophic (Figure 1), especially in the hippocampus and frontal cortex [63,64]. Dystrophic microglia display a small soma (sometimes with apparent cytoplasmic fragmentation due to non-homogeneous protein marker expression) and deramified, shortened, tortuous processes, which may also present spheroid formations [44,51,64]. Dystrophy has often been discussed in relation to senescence-associated states and aging, and has been described to manifest a functional decline with an altered phagocytosis frequently seen in parallel with inflammatory phenotypes (Figure 1) [64]. Region-dependent associations are also apparent with dystrophic microglial distribution. In the presence of disease pathology (i.e., in AD), the majority of microglia have been reported to appear dystrophic in the hippocampal formation (i.e., dentate gyrus, CA1), while such a proportion displayed only modest deviations in other regions in the neocortex [10,44]. Accordingly, microglial dystrophy has been associated with cellular accumulation of ferritin, a key phenotype in aged microglia (Figure 1) [65]. Since microglia can sequester iron and store it in ferritin, researchers proposed that iron-mediated oxidative stress may contribute to microglial damage and cumulatively result in a dystrophic form in aging contexts [63]. Contrary to previous hypotheses, iron accumulation within microglial states does not directly define the electron-dense nature of dark microglia; ultrastructural analysis in mouse brains showed dark microglia populations not staining for non-heme iron and the reverse, typical microglia presenting staining for non-heme iron [66]. Microglial iron overload was observed in AD pathology, relatively heightened than in physiological aging [66], suggesting that microglial iron buildup could be broadly contributing to chronic inflammation and the progression to microglial disease states. Quantitative studies in humans indicate that dystrophic microglia could represent a pathological component instead of an outcome of healthy brain aging, as often observed in disease-associated microglia in AD, as discussed in later sections [10,38,44,61,63,67,68]. To clarify whether this microglial dystrophic state represents physiological aging independently or as a product of disease-associated dysfunction is essential to distinguish physiological aging mechanisms from those that drive AD and related neurodegenerative disorders.

As exemplified in the discussion above, and consistent with spatial influences on microglial molecular states, quantitative post-mortem analyses further support regional differences in the relationship between aging and microglial morphologies. It has been reported that in hippocampal CA1, both hypertrophic and dystrophic microglia increase in absolute number with aging [44]. However, the proportion of dystrophic microglia was shown not to be age-dependent, whereas hypertrophic microglia increase as a fraction of total microglia with advancing age [44]. Additionally, dystrophic microglial proportions in neocortical gray matter correlated with age but not with hypertrophic microglia, emphasizing regional variation in microglial morphological remodeling during healthy aging [44]. These findings indicate the importance of interpreting microglial morphology and its functional implications while considering their regional distribution when evaluating physiological aging trajectories.

All in all, the emergence of hypertrophic, dystrophic, and other stress-associated microglial morphologies in the aged brain could indicate that microglia transition from a homeostatic state and function toward states marked by chronic reactivity and reduced functional capacity. A central unresolved question is whether the aging-related microglial morphologies and cellular stress features represent reversible responses to cumulative insults or instead reflect irreversible aging processes (i.e., cellular senescence). The following section, therefore, focuses on age-related microglial inflammatory states, inflammaging, cellular senescence, and oxidative stress as candidate mechanisms that may couple microglial morphological changes to impaired regulation of CNS inflammation and increased vulnerability to age-related neurodegenerative pathology.

### 2.2. Molecular Drivers of Aging-Associated Microglial Phenotype

By mapping the gene expression landscapes in human microglia, advances in transcriptomic approaches (i.e., RNA sequencing or RNA-seq, and single-cell RNA-seq) have expanded our understanding of microglial states in both aging [18,19] and neurodegenerative diseases like AD [69]. In this section, we explore various microglial molecular changes in the aging brain, highlighting transcriptomic and proteomic shifts that may help to elucidate how microglial programs relate to normal aging and age-related pathology.

#### 2.2.1. Molecular Landscape in Aging Microglia

Age-dependent reduction of expression of several homeostatic transcripts in microglia has been observed in older adults (Figure 1), notably in the human post-mortem brain (cerebral cortex) at 95 years of age [19]. This includes genes encoding for microglia-neuron crosstalk-related receptors (i.e., *P2RY12*), transforming growth factor-β (*TGFB*), interferon, as well as molecules driving cell polarity and motility (i.e., *PFN1*), cell adhesion (i.e., *ICAM3*), and actin assembly (i.e., *TLN1*, *PFN1*, *EVL*) [18,19,40,70], without significant sex dimorphism identified [40]. It was also shown that aged microglia exhibit downregulation of pathways related to IL-6 cytokine signaling and antioxidant defense, most pronounced in the subventricular zone, a region essential for neurogenesis [40]. Essentially, age-dependent changes in the expression of microglial immune-response programs ensue generally across cerebral regions in both males and females: genes associated with inflammation including cytokines, natural killer (NK) cell signaling, immune response regulators, and phagosome formation increase, while genes linked with actin dynamics decrease with aging [28,40,71,72]. Systemic chronic inflammation is a defining feature of aging [10], and these human microglial transcriptomic findings may thus be framed as a shift toward a pro-inflammatory baseline status in aging (Figure 1) [73,74]. In addition, some gene signatures are increased in mouse but decreased in human microglia, or vice versa; hence, some gene signatures are not conserved between mouse and human microglia, underscoring limitations in cross-species translation [19]. Of note, these transcriptomic changes appear to become more prominent starting in late adulthood (sixth and seventh decades of life) in humans and increase in older age [28], at which expression of genes associated with cell senescence in microglia displays age-related abundance [75,76]. Therefore, aged microglia seem to resemble a homeostatic phenotype, but with reduced motility, inefficient intercellular crosstalk, and dysregulated immune response, perhaps becoming more similar to a senescent state. Together, these advances may support a shift to a heightened chronic inflammatory state, coupled with dysregulated immune reactivity, as a prominent feature of human microglial aging [19,77,78].

In this context, to complement transcriptomic signatures, proteomic characterization of microglia aids in verifying key protein–protein interaction and detecting post-translational modifications (e.g., sirtuin 1 (SIRT1)-mediated deacetylation) that constitute the microglial proteome landscape to help comprehensively define the changes in microglial phenotypes across the human lifespan and in disease. For instance, a comprehensive multiplexed mass cytometry (CyTOF) study identified core human microglial markers at proteomic levels in post-mortem male and female human brains spanning adulthood (23–80 years) [79,80,81,82]. Human microglial homeostatic phenotype includes high expression of characteristic microglial markers (e.g., transmembrane protein 119, TMEM119), cell surface receptors, initiation factors of phagocytosis, immunoregulatory factors, and low-to-absent expression of receptors associated with anti-inflammatory regulatory response, at both the transcript and protein levels [18,19,80,81,82]. This proteome profile confirms a pertinent involvement of key transcriptomic signatures in homeostatic human microglia. Moreover, age-related changes in the homeostatic microglial immunophenotype (i.e., protein marker expression) include elevated expression of major histocompatibility complex (MHC) class II antigens [83], which are associated with inflammation and antigen presentation. These changes related to MHCII antigen expression were observed in ramified microglia distributed in both white and gray matter and linked to advancing age [83,84,85]. Conde & Streit (2006) [83] further suggest age-related myelin breakdown may correlate with microglial expression of MHCII, by facilitating the phagocytosis of myelin degradation products and other types of cellular debris [86]. Thus, the increased expression of this marker in aged microglia could suggest a heightened inflammatory state [87], reinforcing chronic inflammation as a hallmark of aging exemplified in microglial aging. Notably, MHCII-expressing microglia, described morphologically to have large, densely stained soma with short, thick processes that cluster around amyloid plaques and neurofibrillary tangles (NFTs), are also observed in the white and gray matter in patients with AD [84,88], which might support a similar heightened inflammatory microglial reactive state in both physiological and pathological aging. While protein expression data is essential to shed light on the functional significance of the identified molecular signatures in the microglia, proteome profiles across human microglial states remain largely uncharacterized. The human microglial secretome, for example, that may play a role in their age-related pro-inflammatory states and of senescence-associated secretory phenotype (SASP), is yet to be outlined. Exploring this microglial molecular feature could advance our understanding of the microglia-mediated mechanisms that drive chronic brain inflammation and their role in the neurodegenerative process. Overall, characterization of microglia at the phenotypic and proteomic levels has been increasingly expanding; however, the human microglial proteome remains incompletely defined [89].

To highlight, microglia’s age-associated molecular signature displays regional heterogeneity and sex differences, in both humans and mice [19,79,81,90]. As an example, observed in mice, the most pronounced microglial molecular changes in aging have been observed in the hippocampus, a region essential for memory function and severely affected with AD-related neuropathology, compared to the cerebellum and other forebrain regions [81,90]. Age-associated remodeling of microglial gene expression has been evidenced by transcriptomic reports from post-mortem human cerebral cortex, it involves biological processes related to immunoregulatory response, metabolic pathways (e.g., decline in protein homeostasis), and a shift away from homeostatic functions, including a decline in inflammatory control, process motility and phagocytic capacity [18,19,40,91]. This transformation is consistent with findings from aging mouse studies [90]. Importantly, microglia in the aging brain show sex-dependent patterns. In rodents, most changes in the microglial transcriptomic aging trajectories are more salient in females than males, such as an increase in abundance of disease-associated microglia (DAM), a transcriptional state associated with decreased homeostatic gene expression and often observed in the presence of pathology (i.e., AD), most pronounced in the hippocampal regions [92,93,94]. Estrogen in females was shown to display anti-inflammatory and regulatory activity in microglia through endogenous estrogen receptors (ER; e.g., ERα, ERβ) [95], and the loss of these overall protective effects during menopause may coincide with the aging-related upregulation of pro-inflammatory signatures in microglia [96]. Thus, this biological process potentially contributes to an increased AD risk and the onset of neurodegeneration in females [96]. Accordingly, through transcriptomic analysis in post-mortem human brains, distinct alterations in female microglia in the cerebral cortex has been shown to exhibit heightened pro-inflammatory upregulation, including a metabolic shift to glycolysis [97]. This change has been suggested to be driven by the induction of the microglial AKT serine/threonine kinase (AKT)-mechanistic target of rapamycin (mTOR)-hypoxia-inducible factor-1α (HIF1α) pathway by C3a complement production, a signaling cascade associated with increased inflammatory activity, hypoxia-related metabolic response, and impaired lipid metabolism [97]. To put this in context of a pathological course, apolipoprotein E 4 (*APOE4*), expressed in microglia, is a strong AD-risk factor and confers disease-related sex-bias for females. Female-enriched and disease-associated microglia (FDAMic), a subpopulation of DAM, were enriched in *APOE4* carriers and thereby correlated with advancing pathology and cognitive decline as reported in human brain datasets [96,98]. Microglia in female AD pathology mouse models and FDAMic subpopulation concurrently have been shown to display reduced phagocytic signaling [93,99]. Consistently, such sex dimorphism patterns are concomitant with increased amyloidosis in the parietal cortex of patients with AD [99]. These reports strengthen the link between microglial alterations and apparent sex-biased risk and progression of AD pathology among females [93,99,100]. Nonetheless, a relatively more detailed mechanistic insight and molecular characterization supporting this postulate are currently derived largely from animal models. The mechanisms that drive aging- and AD-related sex dimorphism in humans, especially as mapped across susceptible brain regions, remain incompletely explored. Even so, these findings underscore the importance of elucidating regional and sex differences in microglial states in aging and age-related disease in humans, which could positively impact the trajectory of therapeutic advancements (e.g., hormonal therapy in women), considering a sex-biased susceptibility to AD.

#### 2.2.2. Alterations in Regulatory Immune Response in Aging Microglia

In microglial aging, inflammatory processes are increasingly amplified, and impairment of regulatory mechanisms that normally constrain these deleterious states may contribute to a sustained chronic inflammatory milieu in the brain. This state could heighten cellular vulnerability to challenges and pathological processes. The gene expression of *CX3CR1*, a microglia-enriched receptor implicated in synaptic remodeling, phagocytosis, and inflammatory control, among other key functions, is downregulated in the aged prefrontal cortex as observed beginning at 60–70 years of age in humans [28,101]. This may contribute to impairing microglial regulation of the CNS inflammatory milieu and confer increased susceptibility to neurodegeneration [28,102,103,104,105]. Additionally, through chromatic immunoprecipitation sequencing (ChIP-Seq) data and protein–protein interaction network analysis, gene expression of master transcriptional regulators (MRs) of microglial lineage specification, consisting of transcription factors that provides upstream control of molecular pathways that direct the specific differentiation and identity of cells, had negative correlation with age in the human frontal cortex [101]. Listed in Figure 1, central MRs that have been studied include runt-related transcription factor 1 (*RUNX1*), interferon regulatory factor 8 (*IRF8*), *PU.1* transcription factor, T-cell acute lymphocytic leukemia protein 1 (*TAL1*), and interferon gamma inducible protein 16 (*IFI16*) involved in hematopoiesis, myeloid/microglial lineage or differentiation, proliferation, and cell growth [19,101]. Thus, it was suggested that their modulated expression in older age could play a role in mediating the onset of microglial replicative senescence in normal aging [101]. Moreover, these microglial alterations occur in parallel or in coordination with the downregulation of TGFβ signaling pathways, crucial for the regulation of homeostatic and reactive immune response in microglia [18,40,106]. Therefore, key transcription factors may causally contribute to the maintenance of regulatory mechanisms in microglia across healthy aging [101]; however, these pathways could become destabilized in time due to a variety of biological factors (i.e., exposure to heightened oxidative stress, susceptibility to chronic inflammation, and disease risks) that accompany aging. This immune dysregulation may thereby establish a baseline state that links physiological aging with neurodegenerative pathology, such as AD [24,86]. Thus, these outcomes underscore the importance of immune regulation in preserving healthy microglial homeostasis over the course of brain aging.

#### 2.2.3. Role of MicroRNAs in Aging Microglia

Post-transcriptional regulation by microRNAs (miRNA, miR) was described to participate in microglial molecular homeostasis during brain aging [107]. A significant number of these molecules are differentially expressed in the aging brain [108], and specific miRNAs are linked with the human microglial network. For instance, upregulation of miR-29a in the superior frontal gyrus and post-central parietal regions of the human brain showed a strong positive association with advancing age and was reported to confer neuroprotective effects from neurodegeneration [28,109]. However, this increased expression with age was correlated with reduced *CX3CR1* expression in microglia, suggesting that miR-29a could be involved in driving a chronic inflammatory state during aging [28,110]. Conversely, in post-mortem tissue samples from the anterior temporal cortex and cerebellum from patients with AD, a downregulation of miR-29a was observed and linked to abnormally high levels of beta-secretase 1 (BACE1) (an enzyme that initiates the generation of Aβ) and Aβ accumulation in vitro [110]. Therefore, in aging, miR-29a may play regulatory and protective roles in microglial immune reactivity against neurodegenerative pathology, such as AD. In the context of AD, other than miR-29a, microglia-specific upregulation of miR-146a in the cerebral cortex and hippocampus of APP/PS1 male mice (a model expressing AD-related amyloid pathology) exhibited potential neuroprotective effects by reducing pro-inflammatory cytokine levels and enhancing phagocytosis against pathology [111,112]. In patients with AD, miR-146a has been reported to be significantly decreased in the cerebrospinal fluid, whereas its expression in age-matched controls was high [113]. These reports may imply that dysregulation or loss of miRNA function during aging in humans could have role in the development of cognitive decline or a neurodegenerative phenotype [114,115,116]. Together, this observed link between miRNA expression and microglial molecular programs (i.e., inflammatory state and phagocytic function) reinforces that microglia-mediated immune changes may further contribute to driving the transition from healthy aging toward pathology. Nevertheless, further investigations are needed to characterize the relationship between miRNAs and human microglial function in normal aging, and to explore their potential as a target for microglia-mediated therapy in neurodegenerative diseases [111,112,117,118].

Collectively, age-related molecular signatures in human microglia may support a model in which aging shifts microglia toward a brain inflammatory state, presenting reduced homeostatic inflammation-limiting mechanisms [18,119,120,121]. Such an inflammatory state could “prime” or establish a background of chronic low-grade inflammation in aged microglia that increases microglial reactive response to systemic and CNS insults [24]. In parallel, age-related impairment of microglial surveillance, migration, and phagocytic clearance, affecting core microglial functions such as myelin turnover, synaptic maintenance and plasticity, neurogenesis, and removal of debris or neurotoxic proteins, may be indicated by the reduction of gene expression related to actin dynamics and motility and phagocytosis-related receptor pathways. To maintain CNS and microglial homeostasis, regulatory mechanisms may partially constrain physiological changes in aging microglia. However, these microglial processes may also undergo age-dependent inefficiency together with persistent immune reactivity, which may promote chronic CNS inflammation and increase vulnerability of the aged microglia to challenges and pathology [18,40,106].

### 2.3. Lipid Droplet-Accumulating Microglia as an Aging Phenotype

Recent advances in single-cell RNA sequencing (scRNA-seq) and single-nucleus RNA sequencing (snRNA-seq) have uncovered a spectrum of reactive microglial states that emerge during brain aging. Lipid droplets, labelled by the lipid droplet surface protein perilipin 2 (PLIN2), are initially described in microglia from aged mice [122] and are therefore commonly referred to as lipid droplet–accumulating microglia (LDAM) (Figure 1) [123]. They have been subsequently reported at higher levels in microglia from aged (>60 years old) human hippocampal samples [124]. LDAM are associated with lipid metabolism and homeostasis [40], potentially related to the phagocytosis of age-associated products of myelin degradation [24], and enriched for pro-inflammatory and stress-associated gene signatures, including macrophage inflammatory protein1α/β (*CCL3/4*), nuclear factor kappa b subunit 1 (*NFKB1*), *IL1B*, and cluster of differentiation 83 (*CD83*) [123]. Defective phagocytosis, increased reactive oxygen species (ROS) production, and increased pro-inflammatory cytokine secretion are also identified as features of these LDAM [63,124]. Notably, the LDAM present AD risk genes such as *APOE*, *GPNMB*, and *TREM2*, which overlap with amyloid-associated microglial signatures [123]. This raises the probability that specific microglial states emerging during aging may be more susceptible to maladaptive shifts that contribute to neurodegenerative pathogenesis. Further work is needed to characterize the prevalence, regional distribution, and functional consequences of these microglial states in the aged human brain [28].

### 2.4. Oxidative Stress, Inflammaging, and Cellular Senescence in Aging Microglia

Oxidative stress has long been implicated in the aging process in humans. In human cortical tissue (i.e., frontal cortex), reduced expression levels of mRNA and proteins associated with synaptic plasticity (i.e., neurotransmitter receptors and intermediates of synaptic vesicle transport) and mitochondrial function were associated with chronological aging, beginning in midlife (40 years of age) [125]. These age-related shifts were accompanied by induction of stress-response pathways and signatures consistent with oxidative DNA damage and reduced base-excision repair [125]. Consistent with this, aged human tissues in other organ systems show higher levels of oxidant-damaged DNA [126,127]; as such, oxidative stress in the nervous system has been linked to reduced regenerative capacity and functional decline [127]. Oxidative stress increases reactive oxygen and nitrogen species (RONS) and promotes macromolecular damage, which can activate cell-death pathways and amplify immune and inflammatory signaling (Figure 1) [128]. Oxidative stress in the CNS microenvironment could arise from an age-related chronic inflammatory setting, a feed-forward mechanism that may sustain chronic inflammation [127,128]. Such a biological relationship is central to the concept of ‘inflammaging’: the chronic low-grade inflammatory state associated with aging, which is linked with multiple age-related conditions, including AD [127,129]. To this account, cellular senescence comes into play with an increasing number of studies showing direct correlations between the emanation of a microglial senescent state and oxidative stress and inflammation [128,130]. Cellular senescence is widely recognized as a hallmark of aging [131,132,133,134]. Oxidative stress, as an extrinsic signal, can initiate cell cycle checkpoint pathways that promote cellular senescence [135]. Importantly, senescent cells remain metabolically active and can secrete a SASP [68], while they become resistant to apoptosis and may persist in tissues over an extended period of time [129]. Consequently, these cells can influence their local tissue microenvironment. In line with this framework, multiple studies report age-associated accumulation of senescent cells across tissues, including the brain, in both mouse models and humans [5,134,136,137,138,139,140].

In the aged human brain, senescence-associated markers are expressed across multiple cell types [141]. In particular, microglia have been described to undergo cellular senescence in aging, with increasing p16-positive cells with age and in the highest proportion in healthy samples from donors of 60 years of age and above (Figure 1) [24,142]. They were also identified to represent the primary cell type to confer susceptibility to senescence in the aging brain, as well as in various disease conditions [5,76,137,138,140]. Consistent with the accumulation of senescence-associated programs late in life, an increased expression of genes in the canonical senescence pathway (CSP) has been reported in microglia across older ages in humans [76]. Expression of CSP genes is considered to represent the process of cell cycle arrest and, thus, is utilized as one of the factors in measuring cellular senescence [143]. This supports that microglia have a proliferative limit, also known as the Hayflick limit for replicative senescence, at which they reach the end of their cell cycle by approximately 80 years of age in humans [38,76]. As a hallmark of cellular senescence, telomere shortening has been implicated in the reduction of proliferative potential of aged microglia in in vitro and in vivo rodent studies, as well as in normal human aging [30,144,145,146]. In the context of aging and pathology, cellular senescence has been linked to glial cell dysfunction through sustained pro-inflammatory cascades, which may help explain age-associated shifts in microglial state and function [129]. A systematic review by Malvaso et al. (2023) described a link between microglial SASP with mitochondrial impairment, ROS generation, and DNA damage in advanced age [30,145], consistent with a positive feedback loop sustaining inflammation and oxidative stress. Additionally, epigenetic mechanisms may participate in this sustained pro-inflammatory predilection with age. In primary cortical mouse cultures, microglial cell expression of *SIRT1*, a class III deacetylase identified to play a role in mitigating chronic inflammation and limiting cellular replicative lifespan [147], has been shown to confer potential protective effects against Aβ toxicity and cellular senescence through inhibition of NFkB signaling [148]. Additionally, deficiency in *SIRT1* mRNA levels in microglia isolated from adult mice was observed in an age-dependent manner, and has been associated with significantly higher *IL1B* (a key inflammatory cytokine) mRNA and protein levels in old compared to young adult mice [148]. Methylation data obtained from human postmortem brain tissue and blood samples verified the aging-related selective epigenetic regulation of *IL1B* transcripts by hypomethylation at two primary CpG sites (cg01290568 and cg15836722) [148]. Methylation at these sites has a negative correlation with aging observed in the human cohort without dementia, as well as those with tauopathy-related dementia [148]. Consistent between mouse and human, *SIRT1* deficiency in aged microglia leads to selective epigenetic regulation, resulting in increased *IL1B* levels in both age-dependent cognitive decline and dementia-related pathology [148,149]. Thereby, aging microglia may exhibit reduced proliferation through replicative senescence, associated with a decline in functional capacity (i.e., maintenance of homeostasis, regulation of immune reactivity, phagocytosis) as depicted in various molecular changes observed in aging (Figure 1) [150]. Collectively, these observations align with broader hallmarks of aging and senescence involving DNA damage responses and oxidative stress, senescence-associated inflammatory response, and epigenetic changes [10,18,42]. Defining the characteristics of microglial senescence and how it alters core microglial functions in homeostatic aging and in contexts of neurodegenerative disease is warranted [16,38,138].

Overall, senescence-associated mechanisms may participate in a cycle that involves chronic inflammation and oxidative stress in the aging brain. Microglia may participate in age-related brain inflammation, inflammaging, as a protective mechanism to maintain homeostasis, to clear damaged tissues, senescent cells, or in synaptic maintenance [41]. Whilst, inflammaging is linked to exacerbation of microglia-mediated oxidative stress, through inflammasome activation and mitochondrial dysfunction, causing further tissue inflammation and fueling oxidative stress. Prolonged oxidative stress can irreversibly damage nucleic acids, lipids, and proteins when antioxidant defenses are overwhelmed as implicated in aging [130,151]. This oxidative damage could influence microglia to become senescent and, consequently, further amplify local inflammation through SASP-mediated autocrine and paracrine signaling and potentially propagating senescence-like states in neighboring cells [129,152]. Senescence may also predispose cells to dysregulated production of RONS, contributing to oxidative damage and further exacerbating an inflammatory state [16,130]. Despite strong conceptual links among cellular senescence, oxidative stress, and inflammaging, the causal relationships tying these processes to microglial dysfunction in human aging remain to be fully elucidated. Moreover, these cellular processes overlap with the described mechanisms in AD pathophysiology, as extensively studied in various mouse models of the disease. It is thus imperative to closely examine microglial molecular, phenotypic, and functional patterns in AD in order to grasp their implication in the quest for an effective and safe treatment.

## 3. Microglia in AD Pathology

Across multiple human GWAS, AD risk variants have been identified in or near genes involved in immune function, particularly in pathways enriched in myeloid cells [153]. In human genetics, microglia-related coding variants, such as those in APOE4 and TREM2 (including R47H), have been reported to increase the risk of late-onset AD [154,155,156]. In particular, TREM2 has been considered as one of the most robust microglia-linked genetic risk factors [154,155]. These GWAS results suggest that microglial biology is linked to AD risk, but they do not give a clear response on whether microglial responses are uniformly protective or harmful across disease stages and the local pathological microenvironment [120]. Observed in both humans and mice, microglia become reactive in response to AD pathology, proliferate, and cluster around plaques to form a barrier that limits plaque expansion and promotes plaque compaction, a pattern associated with reduced neuritic damage [157,158,159]. Nevertheless, microglial response may also lead to harmful effects, including increased secretion of inflammatory factors that can damage neuronal structures and exacerbate tau pathology, as well as the occurrence of synaptic loss through complement-mediated phagocytosis of synaptic material [160].

In vivo imaging in the adult mouse cortex shows that microglial processes are highly dynamic and constantly scan the surrounding tissue. Even in their ‘resting’ state, microglia are highly active and can rapidly respond to local perturbations [161]. In AD pathology, microglia accumulation is consistently observed near amyloid plaques and other sites of local damage in the tissue, which coincides with findings in brain studies in mouse models [120,157]. Beyond microglia, AD is also associated with white-matter and myelin alterations, implicating oligodendrocytes; in vitro, Aβ has been shown to damage mature oligodendrocytes [162]. Overall, both mouse and human studies consistently suggest a nuanced and context-dependent role for microglia in AD, where the net effect depends on how strongly they contain plaques, drive inflammatory signaling, and remodel synapses [120].

### 3.1. Microglial Molecular Signatures in AD

Studies using single-cell and single-nucleus transcriptomics in mouse models and human post-mortem brain tissue support the idea that microglia are heterogeneous and can adopt multiple molecular states instead of one generic “activated” signature [36,69,120]. Conserved in mice and humans, a DAM state has been reported as a transcriptional program characterized by lower expression of homeostatic genes and higher expression of genes linked to lipid handling, phagocytosis, and antigen presentation [36,69,120,163]. In the first description of DAM, immunohistochemistry in human tissue showed DAM presenting intracellular Aβ, consistent with plaque-associated phagocytosis and supporting an association with plaque-adjacent localization [36]. Morphologically, a greater proportion of AD-related DAM has been observed to display dystrophy [36], which remains widely recognized as a feature of physiological microglial aging [10,38]. Underscoring the close phenotypic similarity of aged microglia and some disease phenotypes (i.e., DAM), ultrastructural analyses on post-mortem brains suggest that dark microglia are a subpopulation of DAM, and were found to be abundant in aged post-mortem human hippocampus samples [60]. Dark microglia (and intermediates between typical and dark microglia) have been described in pathological contexts such as AD pathology [61,164]. Their numbers were shown to double in aged post-mortem human frontal cortex samples with AD [61], which may suggest an overlapping microglial stress response state (dystrophic and dark microglia) between aging and AD [60,61,165]. Nevertheless, investigation in AD pathology mouse models supported a two-step activation process in which a later component of the DAM program induction depends on TREM2 [36]. TREM2–APOE pathway was suggested as a regulator that acts as a major switch toward DAM [163] as well as dark microglia [164]. Therefore, studies elucidating the effects of modifying human APOE4 and TREM2 to modulate microglial states may provide useful insights into the development of potentially effective and safe therapeutic strategies [166].

Signals or pathways associated specifically with transitions from homeostatic microglia to early stages of the disease process are yet to be defined [167]. Other studies have described a neurodegenerative microglial phenotype, termed the microglial neurodegenerative phenotype (MGnD), and suggested that this population may have a specific neurotoxic role [163]. The MGnD transition was reported to be dependent on the TGF-β-dependent homeostatic microglial program. As shown in Table 1, both DAM and MGnD showed similarities in specific genes observed during the aging process in mice and in AD progression in humans. Specifically, APOE is involved in lipid transport and cholesterol handling and may influence microglial phagocytosis and inflammatory signaling [168,169]. TREM2 and its adaptor, transmembrane immune signaling adaptor (TYROBP)/DAP12, contribute to damage sensing, microglial survival, migration and phagocytosis [170,171]. C-type lectin domain-containing 7A (CLEC7A, or Dectin 1) is associated with tissue damage recognition and phagocytic/inflammatory activation [167,172]. β-amyloid binds to microglial CLEC7A to induce an inflammatory response in the pathogenesis of AD [172]. Cystatin F (CST7) is linked to lysosomal and phagocytic activity in microglia [173], and integrin subunit alpha X (ITGAX)/CD11c marks microglial populations emerging during aging and in neurodegenerative disease conditions [168,174]. Those findings suggest common neurodegenerative properties of microglia in AD and aging. Of note, while differences might exist in genes affiliated with MGnD and DAM, they might be attributed to neurotoxic microglial changes connected with neurodegenerative processes rather than to specific AD-related pathological changes (i.e., MGnD vs. specific AD-related alterations, such as those in DAM).

Stressed (and dark microglia), also considered a neurodegenerative microglial phenotype, exhibit neurotoxic capacity contributed by their production of toxic lipids [61]. These results may suggest potential pathways that can be targeted in disease progression. Research in human post-mortem brain, using single-cell studies, describes programs similar to the DAM-like or MGnD states identified in AD pathology mouse models, as well as interferon-associated programs linked to neurotoxic microglial states. However, the correspondence between mouse-defined and human-defined states is only partial [120]. Importantly, combined mouse and human data indicate that microglial states cannot be captured by a single biological variable: distinct states likely reflect different local drivers and limitations and may lead to different functional outcomes [120,167]. Thus, comparisons across mouse and human datasets should clearly report tissue source, pathology burden, and cell isolation method, because these technical choices can change which microglia are captured and measured in the dataset. As a result, some microglial states may appear more common, or may not be detected at all, even if the underlying biology is similar [120]. Of note, animal models are designed to reproduce selected features of the disease process in AD, like amyloid or tau pathology, but do not fully reflect the multifactorial, heterogeneous and long-term process of the human disease. In AD, Aβ and tau pathologies might be linked with alterations in the vascular system, metabolism, and hypertension that may impact microglial states and are not necessarily seen in mouse models. Nevertheless, similarities between genes associated with DAM and MGnD observed in both mice and patients with AD suggest that there is a potential relevance in studying microglial changes, their outcomes, and the underlying molecular mechanisms using mouse models linked to pathology. These can also be complemented by studies using humanized models, human cellular models, and human samples. Considering this, we discuss microglial molecular landscapes in humans in conjunction with murine studies in this section to scope microglial molecular changes in AD-like CNS microenvironments.

### 3.2. Microglial Phenotypic and Morphological Alterations in AD

In mouse amyloidosis models, microglia near plaques typically have larger cell bodies with fewer, shorter and thicker processes than homeostatic ramified microglia in non-plaque regions [157,158,159]. Studies using high-resolution confocal and two-photon imaging show the appearance of microglial processes around plaques, forming a barrier that may affect plaque compaction and toxicity [157]. This may suggest a key role for microglia in shaping amyloid plaque size and load [157]. Accordingly, post-mortem studies showing clusters of microglia with deramified and discontinuous ionized calcium-binding adapter molecule 1 (IBA1)-labelled processes, previously characterized as a dystrophic microglial state, intertwined with neuritic plaques [49,51]. It was also shown that microglia in AD pathology exhibit dysfunction-related gene expression [121] with age [179], which may affect their activity and result in increased brain amyloid load and neurotoxicity.

**Figure 2:**
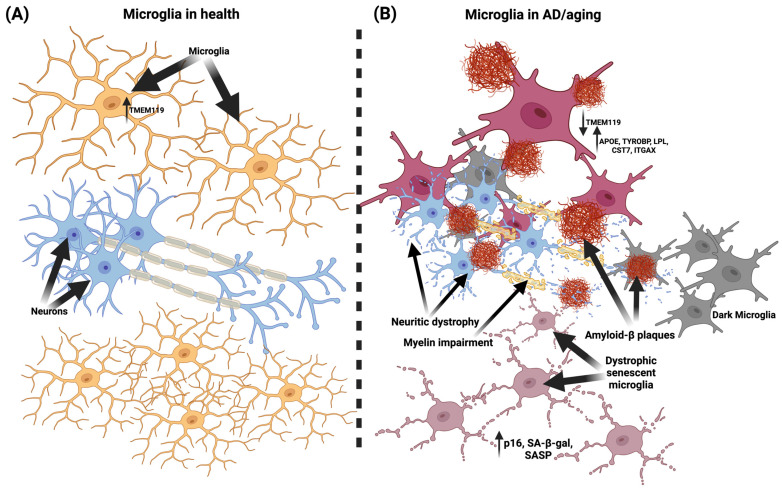
The spectrum of aging and AD-associated microglial states. In the healthy adult CNS, microglia predominantly exhibit homeostatic states (**A**), supporting synaptic homeostasis. In both AD and aging, microglia comprise a spectrum of states, ranging from disease-associated to dark, dystrophic and senescent microglia (**B**). Their relative abundance varies according to the pathological context and disease stage. Alzheimer’s disease (AD); apolipoprotein E (APOE); cystatin F (CST7); cyclin-dependent kinase inhibitor 2A/p16INK4A (p16); disease-associated microglia (DAM); integrin subunit alpha X (ITGAX); lipoprotein lipase (LPL); senescence-associated β-galactosidase (SA-β-gal); senescence-associated secretory phenotype (SASP); transmembrane protein 119 (TMEM119); TYRO protein tyrosine kinase binding protein (TYROBP). Created in BioRender (last accessed on 19 June 2026).

Overall, these changes can be viewed along a spectrum (Figure 2), homeostatic microglia are highly ramified and continuously survey neuronal circuits to support synaptic homeostasis through physiological pruning, whereas in aging/AD plaque-associated microglia (often described within DAM/MGnD-like responses) cluster at plaque borders and contribute to plaque compaction and local responses to neuritic dystrophy; in parallel, dystrophic/senescent-like microglia with seemingly fragmented processes and impaired clearance can emerge away from plaques as disease progresses. At the molecular level, plaque-associated microglia can be distinguished by a shift from homeostatic to a damage-response gene program that includes genes related to lipid metabolism and phagolysosomal pathways [36,163]. This state is typically associated with the upregulation of *APOE* and TYRO protein tyrosine kinase binding protein (*TYROBP*), as well as lipid metabolism and lysosomal genes like lipoprotein lipase (*LPL*) and cystatin F (*CST7*), and integrin subunit alpha X (*ITGAX*; CD11c), which is often used as a marker of plaque-associated DAM/MGnD-like microglia [36,163]. This process shows partial dependency on TREM2, as the initial microglial response to pathology can occur, although some components of the plaque-associated microglia state, including a strong upregulation of CD11c/lipid-lysosomal pathways, are blunted in the absence of TREM2, consistent with the involvement of the TREM2-TYROBP axis in the maintenance of disease-associated microglia [36,158,159,163]. Dystrophic microglia, with distinctive cellular morphology showing “beaded” processes and signs of cellular degeneration, have been found in human AD brain tissue [49].

In both mouse models and human AD tissue, super-resolution imaging and histological analyses show that TREM2-enriched microglial processes accumulate around early amyloid fibrils and plaques, in line with a role in plaque compaction and insulation. In mice, TREM2 haplodeficiency (*TREM2^+/−^*) compromises this barrier function [159]. Early microglial responses driven by TREM2 have also been reported to reduce plaque-associated toxicity, supporting a potential beneficial link between TREM2 signaling and plaque remodeling [158]. Of note, the reported effects of TREM2 signaling on microglial responses and plaque pathology vary across studies and suggest that at later stages, TREM2 may instead be linked to a neurotoxic microglial profile [180]. Nevertheless, TREM2 is being explored as a therapeutic target for modulating microglial state [181]. Functional perturbations further support a plaque-modifying role for microglia [69]. It was reported that parenchymal plaques largely fail to form after depletion and that Aβ deposits shift toward cortical blood vessels, reminiscent of cerebral amyloid angiopathy, with residual plaque-forming microglia exhibiting a DAM-like profile [69,182]. Finally, microglial morphology is shaped by both the microglial state and the surrounding CNS microenvironment. For this reason, reviews that integrate mouse and human data highlight that morphology should be interpreted together with molecular markers and the spatial context, rather than alone when defining the microglial phenotype [69,120].

### 3.3. Microglial Senescence in AD Pathology

Cellular senescence is defined as a state of cell-cycle arrest (often in G1) that results from stress responses and may increase the secretion of SASP factors. This may lead to an increased pro-inflammatory profile of the microenvironment, through the secretion of cytokines (e.g., IL-6, IL-1β, TNF-α), chemokines, and matrix-degrading enzymes [183]. These features align with processes implicated in neurodegenerative diseases, but proving senescence in vivo is still difficult [184]. For this reason, a multi-marker approach has been proposed: rather than relying on one marker, senescence is evaluated through a set of hallmarks, including cell-cycle withdrawal, macromolecular damage, SASP, and metabolic alterations, which are relevant for assessing “senescent-like” microglia [185].

Aβ plaque deposition in human post-mortem brains has been associated with upregulation of genes and pathways linked to double-strand DNA breaks, mitochondrial dysfunction, and ER stress genes and pathways, with these signatures particularly enriched in microglia [76]. These processes may promote a premature senescence response [16,76]. In a mouse tauopathy model expressing MAPT P301S (PS19), senescent astrocytes and microglia positive for p16^INK4A^ were reported to accumulate [186]. Consistent with this, senescence markers, β-galactosidase and p16^INK4A^, were increased more than fourfold and twofold, respectively, in human AD brains [76]. Notably, some mouse and human evidence suggests that cellular senescence in brain cells, predominantly in microglia, may precede the Aβ deposition and tau hyperphosphorylation [75,186,187]. For instance, in mouse models, functional experiments showed that clearing senescent cells reduced tau-dependent pathology and cognitive decline [186], while recent human data further support an association between microglial senescence-related alterations and AD pathology [188]. Another study reported that in an amyloidosis mouse model, early and sustained microglial proliferation promoted replicative senescence, including increased β-galactosidase signal. Furthermore, it was suggested that microglia in an AD pathology mouse model acquire an inflammatory, neurotoxic DAM profile that may contribute to early Aβ pathology [145]. Therefore, senescent microglia may be both the result of Aβ pathology and also accelerate AD pathogenesis and progression [76]. It was also recently reported that AD-associated senescence signatures can be found in different CNS cell types, with microglia exhibiting the most perturbed senescence-related profiles, resulting in phagocytic, lipid-processing, and inflammatory states [188]. Furthermore, transcriptomic analysis in human microglia showed that downregulation of phagocytic pathways and upregulation of senescence-related genes were associated with greater Aβ load [76,163,189]. This corresponds to the perceived dysfunction in microglia linked with AD, which could impact microglia’s role in maintaining brain health and homeostasis [190,191]. It was thus hypothesized that dysfunctional microglia interacting with AD pathologies become senescent [16], while this proposition needs further investigation.

Although cellular dystrophy is not considered conclusive of senescence, some studies suggest a “senescent-like” interpretation when combined with the presence of other senescence markers. Therefore, senescence is considered to be a multi-marker concept, including cell cycle regulators such as p16INK4A/CDKN2A, lysosomal SA-β-gal activity, and SASP, rather than any one of these features being considered conclusive [49,184,185]. In human neuropathology, dystrophic microglia with seemingly fragmented or beaded processes and signs of cellular degeneration have been described in the AD brain, and Braak-stage analyses have been used to examine the association between microglial dystrophy and the severity and spread of tau pathology [49]. Furthermore, the same human study reported that Aβ deposits lacking tau-positive structures colocalized with ramified microglia, consistent with the idea that Aβ alone is not always sufficient to produce the classic histological ‘activation’ profile of microglia as defined by their criteria [49]. However, morphology alone is not enough to demonstrate cellular senescence in human tissue, and this is why senescence reviews recommend using multiple markers and functional context for reliable in vivo identification [184,185]. Taken together, the strongest evidence that senescence-like mechanisms may contribute to AD-related phenotypes comes from animal studies that combine multiple markers with functional interventions, whereas human studies often rely on morphology or transcriptomic signatures that should be interpreted cautiously [49,120,185,186].

## 4. Therapeutic Implications: Emerging Strategies Targeting Microglia in AD

Microglia exhibit a context-dependent role in the pathology of AD. Physiologically, microglia play an essential role in maintaining brain homeostasis; however, ineffective or dysfunctional microglial programs could foster chronic brain inflammation, impair microglia-associated mitophagy, exacerbate Aβ-amyloid deposition and tau pathology, and accelerate neuronal and synaptic loss, resulting in cognitive decline [192,193]. Evidence indicates that disease-associated microglial states are characterized by reduced phagocytic capacity, cellular senescence, and sustained pro-inflammatory signaling [194]. These molecular events could thereby reflect inadequate immune adaptation at the early stages of the disease and excessive innate immune response during the later stages, and subsequently amplify the disease pathology in AD. In addition, cellular alterations indicating senescence are found in post-mortem brain samples of patients with AD and in mouse models [195]. Particularly, microglial senescence intensifies neuronal dysfunction by impairing Aβ clearance, promoting chronic neuroinflammation and upregulating SASP secretion [188]. Therefore, targeting senescent microglia offers a promising treatment strategy to alleviate AD pathology by reducing SASP-driven neuroinflammation and reestablishing microglial function. Hence, senolytic and senomorphic interventions may consequently mitigate AD pathology and allow for the delay or halt of the resulting cognitive decline.

Despite extensive efforts to modify disease progression, effective clinical translation remains elusive. Table 2 synthesizes the current strategies targeting microglial functional states, through the modulation of microglial reactivity, immunoreceptor signaling, anti-inflammatory strategies, or replacement of dysfunctional microglia, which are promising therapeutic avenues for mitigating AD progression.

### Pharmacologic Modulation and Immunotherapeutic Approaches Targeting Microglia in AD

Therapeutically replenishing functional microglia can mitigate AD pathologies [196]. For instance, stem cell-based therapy, including transplantation of microglia-like cells derived from human induced pluripotent and embryonic stem cells [197,198], could be a favourable strategy to restore microglial function. Transcriptionally, these stem cell-derived microglia are similar to human fetal microglia, which demonstrate robust phagocytic activity and rapid responses against pathological stimuli [199,200], analogous to homeostatic microglial states [201,202]. However, this therapeutic approach may have significant limitations, including low throughput and variability in engraftment. To overcome these limitations, there are emerging ideas suitable for disease modelling and drug testing, such as integrating human pluripotent stem cell (hPSC)-derived microglia (IMG) into cerebral organoids to create a unique, human-related, multidimensional microenvironment [203,204]. Furthermore, AD-risk genes encoding microglial immunoreceptors have been identified through GWAS. Among these, *TREM2*, a myeloid-expressed lipid and lipoprotein-binding receptor, and *CD33*, a sialoadhesin molecule and immunoglobulin-related myeloid receptor, emerged as the most strongly associated with AD and, thus, extensively studied [205].

**Table 2:** Therapeutic strategies targeting microglia- and immune-related molecular processes in Alzheimer’s disease (AD). This table systematically provides investigational agents for AD, organized by therapeutic class, mechanism, preclinical rationale, clinical trial results and identified constraints. Interventions directly targeting microglial function (i.e., TREM2 agonist, AL002) corroborated favourable effects in vitro and in vivo on plaque and neuroinflammatory pathology, yet failed to hinder disease progression or cognitive decline in Phase 2 trials, with reported safety concerns. Similarly, repurposed anti-inflammatory drugs, montelukast, pioglitazone, and minocycline, all demonstrated compelling preclinical or epidemiological signals, but yielded no consequential cognitive or biomarker benefits in randomized controlled trials, due to inefficacy, adverse effects or disease-stage dependency. Only amyloid-targeting monoclonal antibodies such as lecanemab and donanemab substantiated consistent, but modest reduction of cognitive impairment in early AD, weighed against notable ARIA menaces. Novel immunomodulatory biologics (i.e., Xpro1595) have demonstrated initial promise in reducing CSF and MRI inflammatory markers in Phase 1, but larger Phase 2 validation is pending. Complement inhibitors and emerging approaches, including CRISPR, engineered or modified immune cells, and vaccine adjuvants, remain utterly preclinical, with technical obstacles involving off-target effects and lack of cell precision. The table collectively highlights an indispensable field-wide challenge, due to the inability of potent preclinical anti-inflammatory approaches to translate into clinical significance, except for the amyloid-directed immunotherapies with marginal benefits and mainly in males. Arrows broadly describe changes in molecular level, biological process, function or other factors specified: (↑) indicates an increase and (↓) indicates a decrease. Triggering receptor expressed on myeloid cells 2 (TREM2); cluster of differentiation 33 (CD33); amyloid-beta (Aβ); Alzheimer’s disease (AD); peroxisome proliferator-activated receptor gamma (PPARγ); Mini-Mental State Exam (MMSE); NOD-like receptor family, pyrin domain-containing 3 (NLRP3); tumor necrosis factor (TNF); cerebrospinal fluid (CSF); mild cognitive impairment (MCI); magnetic resonance imaging (MRI); complement component 1q (C1q); Janus kinases (JAK); signal transducer and activator of transcription proteins (STAT); p38 mitogen activated protein kinase (p38 MAPK); programmed cell death protein 1 (PD-1); clustered regularly interspaced short palindromic repeats (CRISPR).

Type/Action	Drug Name	Experimental Outcomes	Findings in Clinical Trials	Identified Constraints	References
**TREM2 agonist**	AL002	From Phase 1 in vitro and in vivo data: ↓ Filamentous plaques [206] ↓ Neurite dystrophy [206] ↓ Microglia inflammatory response [206]	INVOKE-2 Phase 2 trial in patients with early-onset AD: No clear effects on disease progression nor cognitive decline [207]	Could pose immune adverse effects [208]	[206,207,208]
**Anti-CD33 antibody**	Lintuzumab	↑ Aβ phagocytosis [209] ↓ CD33-mediated neurotoxicity	-	Not yet tested clinically	[209]
**Cysteinyl leukotriene receptor antagonist**	Montelukast	Human cohort data: ↓ Cognitive decline [210] ↑ Preserved functions in non-amnesic cognitive domain in AD dementia [210]	Phase 2 trial in patients with MCI and early AD: No clear biomarker or cognitive benefits over one year New Phase 2 trial in patients with mild to moderate AD: Completed enrolment but with results still pending [211]	AD fly and mouse models: Disease stage dependent [212]	[210,211,212,213]
**Non-steroidal anti-inflammatory Drugs (NSAIDS)**	e.g., Naproxen	From epidemiologic studies: ↓ risk of AD or delayed onset [214]	INTREPAD trial with Naproxen in elderly patients with AD family history and without cognitive disorders: No effect on disease progression Reported adverse effects [215]	In randomized trials: No cognitive or disease benefits May pose safety risks [214]	[214,215]
**PPARγ agonist**	Pioglitazone	↓ Pro-inflammatory genes expressed in microglia [216]	TOMORROW Phase 3 trial with cognitively healthy participants aged 65–83, at high and low risk of developing AD: Discontinued due to lack of efficacy [217]	In early AD patients: Mixed results in improving cognitive function [216]	[216,217]
	Cannabidiol and other cannabinoids	In vitro: modulates microglia reactivity [218] In mice: neuroprotective of memory [218]	Phase 2 trial in patients with AD-associated dementia, aged 60–80: Improved cognitive function based on MMSE No reported adverse effects [219]	Larger and longer trials required to confirm effects [219]	[218,219]
**NLRP3 inflammasome modulating agents**	Minocycline	↓ Inflammation [220] ↓ Microglial recruitment [220] ↓ Aβ burden [220]	2-Year randomized clinical trial in patients with mild AD: No significant cognitive benefits in patients [221]	-	[220,221]
	Edaravone	In vitro: ↓ Mitochondrial dysfunction [222] ↓ Aβ induced microglia reactivity [222]	-	Clinical relevance remains to be proven	[222]
**Immunomodulatory Approaches**					
**Amyloid targeting monoclonal antibodies (mAbs)**	Lecanemab, Donanemab	↓ Aβ markers in early AD [223] ↓ Decline of cognitive function [223]	Phase 3 trial of lecanemab in patients aged 50 to 90 with mild AD: Reduced Aβ markers and cognitive decline [223] Phase 3 trial of donanemab in symptomatic patients with early AD: Slowed disease progression [224]	Only ↓ disease progression by 20–30% [223] Serious side effects (ARIA) [225]	[223,224,225]
**TNF inhibitors**	Etanercept	↑ Cognition in one patient with AD [226]	Phase 2 trial in patients with mild to moderate AD-associated dementia: Subcutaneous administration was well-tolerated [227] No statistically significant changes in cognition, behavior or global function [227]	Larger trials including placebo group still missing [227]	[226,227]
	Adalimumab, Infliximab	↓ AD and dementia risk in large inflammatory cohorts	Trials specific for AD are limited	Not established causality	[228]
	Xpro1595	↓ Inflammatory activity without impeding essential TNF functions [229]	Phase 1 trial in 20 patients with AD: ↓ CSF inflammation and neurodegeneration biomarkers and MRI inflammation markers [229] Phase 2: To be held evaluating cognition and plasma markers in a larger cohort [230]	-	[229,230]
**Complement inhibitors**	C1q antagonists	↓ Maladaptive microglia-mediated phagocytosis and synaptic pruning [231,232]	-	-	[231,232]
**Other immune-modulating agents**	JAK/STAT pathway modulators [233], p38 MAPK inhibitors [234], PD-1 inhibitors [235], lenalidomide [233], galectin-3 antibodies [236]		Advancing through clinical trials	Efficacy is yet to be established	[233,234,235,236]
**Emerging strategies**	CRISPR-based		Lack cell-specificity and have off-target effects [237]		[237]
	Engineering immune cells to ↑ Aβ clearance, modulate regulatory T-cells, repurpose vaccine adjuvants to induce protective microglial phenotypes [238,239]		-	-	[238,239]

Since the discovery of TREM2, substantial efforts have been invested in understanding its biology and involvement in shaping microglial response in AD. For example, preclinical studies demonstrate that several TREM2 agonistic antibodies enhance microglial responsiveness to Aβ and attenuate amyloid pathology, conferring neuroprotective effects [240,241]. Supporting the translational potential of TREM2-targeted immunotherapy in AD, favourable tolerability in a Phase I clinical trial was reported for the clinical candidate AL002, derived from the preclinical antibody AL002c (Table 2), and shown to reduce filamentous plaques and neurite dystrophy, and tampered microglial neuroinflammatory response [206]. These reports encouraged the progression of a randomized, double-blind, placebo-controlled study (INVOKE-2) for patients with early-onset AD, a global Phase 2 trial. However, AL002 did not achieve the anticipated outcomes of slowing clinical progression of symptoms or improving cognition, and was subsequently discontinued [207].

Unarguably, CD33 polymorphisms are implicated in AD susceptibility and pathology, with the CD33 sialic acid-binding domain mediating the suppression of microglial phagocytosis of Aβ, thereby making it a potential therapeutic target in AD [205]. CD33-targeting antibodies are investigated, which could potentially inhibit CD33-mediated neurotoxicity [242]. Notably, lintuzumab, an anti-CD33 antibody (Table 2), utilized for the treatment of acute myelogenous leukemia, has been suggested for repurposing in AD, although the feasibility remains hypothetical and well-defined clinical evaluation is necessary. Additionally, a quantitative binding assay demonstrates that CD33 binds strongly to clusterin but not to ApoE, indicating that it acts as a sialylation-dependent ligand. Co-immunoprecipitation experiments additionally reveal co-localization of CD33 and clusterin on microglia in the brains of patients with AD, particularly near Aβ plaques, with evidence that sialylated clusterin alone inhibits Aβ uptake from monocytes of high-risk “CC” carriers and UP937 cells. However, the interaction between clusterin and Aβ induces stronger CD33 ITIM signaling than clusterin alone, facilitating the recruitment of other downstream players such as SHP-1 [242]. Also, when a subtype-selective sialic acid mimetic (P22) is conjugated to microparticles, Aβ phagocytosis is enhanced in a CD33-dependent manner [209]. Overall, these findings identify CD33 and its associated signaling pathways as critical therapeutic targets in modulating microglial reactivity and Aβ clearance in human AD pathology.

Montelukast, a cysteinyl leukotriene receptor antagonist, has also been investigated for mitigating AD pathology, CNS inflammation, and enhancing microglial homeostasis and neuronal viability, although in a disease-stage-dependent manner (Table 2) [212]. Pharmacoepidemiologic reports demonstrate that use of montelukast is associated with reduced dementia risk in AD in large cohorts, and non-amnestic cognitive domains are preserved [210,213]. A Phase 1 study utilizing a dissolving oral film with this drug showed enhanced bioavailability and blood–brain barrier penetration of this drug [243]. In a Phase 2 randomized trial (NCT03991988) in mild cognitive impairment (MCI) and early AD, for over one year, montelukast was well-tolerated but did not show clear cognitive outcomes or biomarker benefits [211]. Nevertheless, another Phase 2 study utilizing a buccal film formulation (NCT03402503) in mild to moderate AD is underway; this trial recently completed enrollment, with global cognition as the primary endpoint.

Importantly, therapeutic approaches that dampen excessive microglial reactivity and enhance their phagocytic capacity may potentially alleviate the heightened inflammatory state associated with AD. Previous long-term epidemiologic studies show that chronic non-steroidal anti-inflammatory drugs (NSAIDs) use is associated with a lower risk of AD (Table 2); in contrast, randomized trials in MCI and AD have shown no cognitive benefit and some negative outcomes [214]. A large trial, Alzheimer’s Disease Anti-Inflammatory Prevention Trial (ADAPT), revealed that NSAIDs are not associated with a reduction of AD incidence or slow cognitive decline, and may pose a safety risk; naproxen, for instance, showed weak or delayed indication of benefit [215]. Overall, meta-analyses indicate that nonspecific anti-inflammatory strategies may not confer an effect in AD, which is attributed to late intervention, short therapeutic exposure, and poor immune-target specificity. In this context, NSAIDs are involved in the activation of peroxisome proliferator-activated receptor gamma (PPARG), a transcription regulator that works to suppress the expression of pro-inflammatory genes in the microglia, which was proposed to be neuroprotective and, thereby, remains a mechanism of interest with its potential therapeutic application [216,244]. This led to a clinical evaluation of the PPARG agonist, pioglitazone, in AD through the TOMMORROW trial (AD-4833; NCT01931566); however, phase III trials were discontinued due to the lack of efficacy and convincing clinical benefits [217]. Moreover, cannabidiol-induced activation through PPARG is also reportedly linked to enhanced neurogenic activity in the hippocampus [244]. Endocannabinoid systems increase with age, and activation of cannabinoid receptor (CB) 1 and 2 receptors is shown to reduce oxidative stress (Table 2). Contrastingly, mouse studies indicate that neuroinflammation is mediated by CB1 and CB2 receptor activation in older age groups [245]. Nonetheless, a recent clinical trial conducted over 26 weeks, patients treated with cannabis demonstrated significantly higher Mini-Mental State Examination (MMSE) total scores than the placebo, although larger and longer-duration trials are essential to confirm these findings [219].

Microglial inflammasome activation, especially the NOD-like receptor family, pyrin domain-containing 3 (NLRP3) complexes, strongly contributes to AD pathology. The NLRP3 inflammasome, which comprises NLRP3, apoptosis-associated speck-like protein containing caspase recruitment domains (ASC), and procaspase-1, drives microglial pro-inflammatory signaling, and its pharmacological or genetic inhibition attenuates neuroinflammation [246]. Thus, these outcomes motivated clinical interest in the potential of inflammasome-modulating agents for AD (Table 2). Minocycline, a blood–brain barrier (BBB)-permeable tetracycline with anti-inflammatory properties, were reported to decrease Aβ burden and microglial response, potentially through NLRP3 inhibition [220]. However, clinical trials for these therapeutic agents failed to demonstrate a significant cognitive benefit in AD patients [221]. Similarly, edaravone, a free-radical scavenger, was shown to suppress Aβ-induced microglial reactivity by inhibiting NLRP3 signaling-mediated IL-1β secretion and mitochondrial dysfunction [222], but its clinical relevance in AD remains unproven. Moreover, purinergic P2X7 receptor is upregulated in plaque-associated microglia in AD brains and acts synergistically with NLRP3 to amplify inflammatory responses, with which, upstream regulators of inflammasome activation, such as the purinergic P2X7 receptor (P2X7R), are thus also implicated [247,248]. Despite promising the preclinical data against AD, clinical trials targeting microglial inflammation have produced very limited success, reflecting the complex, interconnected, and stage-dependent nature of microglial inflammatory pathways, by which a single-target therapeutic agent may not adequately address.

Immunomodulatory strategies also provide avenues beyond therapy with monoclonal antibodies (mAbs) against Aβ (Table 2). Specifically, amyloid-targeting mAbs, such as donanemab and lecanemab, employ immune-related mechanisms to dampen AD pathology, including Fc receptor-mediated microglial phagocytosis and endosomal/lysosomal degradation of Aβ [249,250,251]. This translates to statistically significant reductions in Aβ markers in early AD and a moderate reduction in cognitive decline, yielding a 20–30% reduction in disease progression [223,224]. However, Aβ-directed immunotherapy has potential deleterious effects in microglia, such as a sustained and enhanced antigen-presenting inflammatory state in a dose-dependent effect when tested in a mouse model [252]. These could exemplify the dual protective and pathogenic roles of immune responses in AD, where a precise regulation of microglial inflammatory programs is imperative. Targeting accumulated amyloid, a single protein in a multifactorial disease lasting for decades, is biologically limited. By the time symptoms manifest, amyloid may be a tombstone, not the primary driver. Lecanemab and donanemab achieved sturdy amyloid plaque clearance on positron emission tomography (PET) imaging and a gradual decline by approximately 27–36% over a period of 18 months on the clinical dementia rating–sum of boxes (CDR-SB), a composite measure of dementia severity used in quantifying treatment outcomes and monitoring disease progression, but absolute benefits are minute and approximately 2–3 points, frequently below clinically significant thresholds [253]. Benefits are restricted to early-stage, low-tau patients, representing plaque removal alone as incremental improvements rather than a breakthrough [254]. Multi-target strategies are necessary for overall success. Moreover, these drugs are reported to be associated with serious side effects like microhemorrhages, cerebral edema, and amyloid angiopathy, collectively referred to as ARIA-Aβ-related imaging abnormalities [225]. These typically arose from vascular vulnerability during amyloid clearance [255]. Patients treated with Donanemab also exhibit brain volume loss [256]. It is worth noting that the risk of developing mAb-induced ARIA is higher for *APOE4* (a major regulator of CNS inflammation) carriers who are genetically predisposed to late-onset AD. This AD-risk gene is associated with increased recruitment of perivascular macrophages and peripheral monocytes through a complement-dependent mechanism, thus increasing the risk of ARIA with mAb therapy [257,258,259].

Additionally, an epidemiological study on the clinical use of inhibitors of tumor necrosis factor (TNF)-α, implicated in microglia-related pro-inflammatory disease state, investigated their potential for immunomodulation in AD, which revealed an association with decreased incidence of AD [260]. Etanercept, administered perispinally, is reported in an open-label pilot study to show cognitive improvements in small cohorts of patients with AD, but lacks a placebo control and has not been replicated in rigorously designed trials [226,227]. A placebo-controlled, randomized double-blind Phase 2 trial of weekly subcutaneous etanercept (50 mg) in mild-to-moderate AD showed that the drug was well-tolerated but did not significantly improve cognitive behaviour over 24 weeks [227]. Additionally, TNF inhibitors, including etanercept, adalimumab, and infliximab, were analyzed observationally and retrospectively, and they suggest that their use is associated with a lower risk of AD or dementia in large inflammatory disease cohorts, but these associations do not establish causality and clinical trials specifically designed to assess efficacy in AD are limited. The recently concluded early-phase studies investigated the novel therapeutic agent called Xpro1595, a selective soluble TNF (sTNF)-neutralizing antibody that preserves membrane-bound TNF signaling and is described to limit inflammatory activity without impairing essential TNF functions. Briefly, its Phase 1 trial (NCT03943264) in 20 patients with AD consisted of subcutaneous injections of Xpro1595, which were well tolerated, and showed reduction of cerebrospinal fluid (CSF) inflammatory and neurodegenerative biomarkers, as well as magnetic resonance imaging (MRI) markers of CNS inflammation [229]. Also, an open-label extension and Phase II trials (NCT05318976) are in motion to evaluate its effects on cognition and plasma AD biomarkers in larger cohorts [230].

Dysfunctional microglial phagocytosis and synaptic pruning are shown to be restrained through the action of complement inhibitors, particularly complement component 1q (C1q) antagonists (i.e., BMS-984923, ANX005) and C3 inhibitors [231,232]. This may help to maintain synaptic integrity despite Aβ pathology, indicating that targeting these components in the innate immune signaling pathway could be an effective therapeutic approach. Concurrently, various modulators of immune response pathways, including the Janus kinases (JAKs)/signal transducers and activators of transcription (STATs) pathway (i.e., sargramostim, NCT04902703; baricitinib, NCT05189106), are advancing through clinical trials [233]. Other examples of currently studied immunomodulators targeting microglial programs are p38 mitogen activated protein kinase (p38 MAPK) inhibitor (MW150, NCT05194163) [234], programmed cell death protein 1 (PD-1) immune checkpoint inhibitor (IBC-Ab002, NCT05551741) [235], the immunomodulatory small molecule lenalidomide (NCT04032626) [233], and the galectin-3 antibody TB006 (NCT05476783) [236]. These reflect broad efforts to recalibrate neuroimmune signaling in AD [233,234,235,236]. However, definitive clinical evidence of efficacy is still lacking [261]. Technological advancement using clustered regularly interspaced short palindromic repeats (CRISPR)-based genome editing also offers the potential approach to modify pathogenic microglial risk genes such as *CD33*, *APOE*, and *TREM2*; however, cell specificity in microglia and off-target effects pose a challenge [237,262]. Repurposing vaccine adjuvants, such as protollin, is also being studied to induce protective microglial phenotypes, while additional immune approaches include bioengineering other immune cells to enhance Aβ clearance, and modulation of regulatory T cells to rebalance neuroimmune responses [238,239].

Collectively, immunomodulatory therapies, comprising the majority of clinical trials in AD, hold substantial promise for AD; however, acute and long-term safety risks warrant due consideration. Use of TREM2-targeting antibodies showed immune-related adverse events in clinical reports, including anemia (DNL919) and severe ARIA [208]. These suggest possible drug-induced changes in vascular and immune responses in *APOE4* homozygotes (AL002) [207], which resolve when administration is discontinued. Accordingly, careful monitoring and filling the research gaps are warranted to untangle the immediate treatment-related reactions and longer-term risks, such as infection or malignancy, before clinical implementation. Furthermore, AD-risk genes expressed in microglia, such as *APOE*, *TREM2*, *CD33*, *GRN*, and *IL1RAP*, significantly influence susceptibility to pathology, confirming microglia as modulators of Aβ and tau immunotherapies through Fc receptor-mediated mechanisms, making them a key therapeutic target in AD [263]. Notably, as of 2024, immune-directed therapeutics outnumbered amyloid-targeting agents in the AD pipeline [264], reflecting growing optimism driven by strong genetic validation of immune targets and the diversification of mechanisms beyond single-target anti-Aβ approaches. However, despite strong genetic validation, CNS drug discovery remains particularly challenging due to additional barriers in target validation and lead development compared with peripheral diseases [265].

All in all, microglia exhibit a broad and dynamic spectrum of functional states, governed by complex interrelated molecular pathways, by which modulation of single individual markers could yield paradoxical, incomplete or stage-dependent effects. For instance, TREM2-dependent signaling was correlated with amplified inflammation, while dampening this effector mechanism could hinder debris clearance by microglia; exemplified in AL002 TREM2 agonism causing desensitization and off-target neuronal effects [207]. Clinical outcomes in AD, especially in advanced stages, could also be generally described by its multi-system components (i.e., neuronal, glial, vascular aspects), and that monolithic therapies might oversimplify this complexity. As such, monotherapies could potentially interfere with beneficial microglial physiological and immunological roles or significantly fail to slow down clinical deterioration [207]. Of note, more than 2700 trials for AD have been unsuccessful, with only three approvals thus far [266]. It is now increasingly advocated to employ a combination approach to therapy. In AD pathology mouse models, combination drugs (i.e., letrozole + irinotecan) targeting neuronal and glial pathological clusters, respectively, outperformed monotherapies by reversing changes in tau and Aβ, as well as memory deficits [267]. Essentially, systems-level approaches could effectively address pathway redundancies and disease dynamics yet necessitate advanced modelling and adaptive trials. Notably, contemporary industry and regulatory frameworks favour single-agent development, exhibiting defined primary targets, straightforward pharmacokinetics and streamlined safety profiles, making multi-agent therapies financially and logistically difficult despite the emergence of network pharmacology infrastructure. The transition to multi-modal strategies demands paradigm shifts in regulatory science.

## 5. Perspectives and Future Directions

The global human population is increasingly aging, possibly due to improvements in healthcare. Paradoxically, this increased aging increases the risk for age-related neurodegenerative disorders, including the highly prevalent AD. Despite decades of research efforts, effective disease-modifying therapies to abrogate or delay AD progression seem to remain elusive, highlighting the urgent necessity to decipher the biological aging mechanisms that contribute to the disease onset and progression. Recent GWAS suggest that microglia are central to the biological processes underlying both healthy and pathological aging. Age-related alterations in microglial function are multifaceted yet intricately coordinated, resulting in chronically sustained inflammatory processes and deleterious outcomes. These changes produce a continuum of microglial states that exhibit overlapping but distinct metabolic, epigenetic, transcriptional, morphological, and functional properties during aging, and with pathological features such as Aβ and tau [268,269,270,271]. Clinical trials involving drug classes that target microglia and brain inflammation, and Aβ clearance have not produced satisfactory results, despite promising preclinical outcomes. One of the main challenges appears to be the timing of the intervention, suggesting that the success of a single-bullet microglia-focused intervention is greater in early AD stages, when all the drivers of the complex inflammatory programs are not fully set in motion. However, the effect of this approach may be limited at advanced AD stages, where multiple molecular systems have taken place and could lead to paradoxical outcomes. This is partly attributed to the unavailability of specific clinical microglial state markers. Once such markers become available, we can better assess microglial roles during aging and their transition from MCI through different stages of AD and evaluate interventions tailored to specific disease stages with limited drug-induced adverse effects.

In the absence of specific clinical microglial markers, genome-wide approaches have generated extensive information on the transcriptomic and epigenetic reprogramming at microglia across different disease stages. While this approach often suggests simultaneous, interconnected biological activities, current research trends may often prioritize the most characterized cellular features or molecular targets. This reductionist approach can be beneficial if the major driver of microglia’s biological system can be specifically targeted for therapeutic interventions; however, it makes it challenging when the diverse interconnected pathways, possibly directed by multiple drivers, are subsequently integrated into complete mechanisms that drive microglial (physiological or pathological) molecular and cellular networks. As such, a shift in approach is necessary: a holistic examination of microglial signatures and connection patterns comprising metabolic, epigenetic, transcriptional, morphological, and ultrastructural properties, as well as functional outcomes, over the course of aging, across disease stages and in response to treatment interventions. Achieving this would require meticulous disease stage-resolved or trajectory-based reconstruction of distinct microglial states such that multi-omics profiling (e.g., transcriptomics, epigenomics, metabolomics, proteomics) is integrated with morphology and ultrastructure to determine functional networks (gene regulatory, metabolic flux, ligand–receptor interactome, plaque (or neuron)–microglia communication), per microglial state. This integration is best accomplished in situ, where each microglial cell and its internal and local environments can be studied, with the advantage of providing spatial information on the brain architecture regulated and remodeled by microglia. Such an approach could lead to a cocktail of interventions, especially at advanced disease stages, but also aimed at prevention within the perspective of lifestyle medicine, by focusing on different networks for microglial state normalization.

## 6. Conclusions

The key microglial features observed in the aged brain include hypertrophic or dystrophic forms and molecular alterations linked with reduced homeostatic signatures, impaired physiological functions, heightened oxidative stress, sustained chronic inflammatory background, and senescence-like states. These microglial changes (i.e., morphological and transcriptional) could reinforce a dysfunctional course of microglial aging properties, broadly comprising a decline in their critical roles in neuroprotection and homeostatic maintenance, reduced trophic support and phagocytic function and thereby reduced role in synaptic preservation and remodeling, altered cellular metabolism leading to energy deficit, exacerbated cellular stress and senescence, and a resulting dysregulated immune reactivity. These could overlap with a toxic and reactive microglial profile associated with AD-related TREM2 signaling that sustains neuroinflammation. Hence, human aging presents with various alterations in microglial biology that may shape their vulnerability, through the exposure to modifiable risk factors, to a neurodegenerative trajectory like in AD. Accordingly, the impact of microglial dysfunction on overall brain inflammation including neuronal health underlying behaviour and cognition underscores the potential of microglia-targeted therapeutic strategies to modulate AD onset and progression. Therefore, a definitive mechanistic understanding of the microglial aging process across aging trajectories and transitions toward AD remains a critical research avenue.

An extensive profiling of human microglia in aging and disease is still constrained. Complete proteomic, post-translational/epigenetic, and morphological characterization that captures the aging microglial states dynamic nature, while considering the complex human neurological systems and lifestyle elements (e.g., sleep, stress, exercise, diet and the gut–brain axis) contributing to age-related neurodegeneration, are yet to be attained. Microglial ultrastructure, morphology and molecular states, as well as functional properties, are also often studied in isolation. Highlighting these challenges, one fundamental gap in current studies is the systematic mapping of microglial structural and molecular changes to their functional alterations within several biological levels and in tissue-dependent contexts (i.e., brain regions, sexual dimorphism, genetic vulnerabilities, modifiable risk factors, stage of pathology). The future development of radiotracers to image particular microglial states in humans using PET scan is expected to significantly advance this understanding of microglial age-related alterations and functional outcomes, when combined with MRI approaches to study various elements of the brain architecture regulated and remodeled by microglia. These insights could pave the way for the development of targeted approaches acting on microglial states to delay or halt the progression of AD. Future studies must therefore prioritize the employment of combined approaches, including longitudinal PET and MRI in humans, combined with single-cell RNA sequencing (scRNAseq), single-nucleus RNA sequencing (snRNAseq), and other in situ or spatial molecular strategies, together with morphological and ultrastructural analysis, in human tissue samples, human cellular models and humanized mouse models. This will allow to characterize the aging process of human microglial states, and identify transitions toward age-related neurodegenerative disease conditions such as AD. Ultimately, this knowledge of microglial biology could help in crossing the unknowns in microglial state normalization as a therapeutic target, to hasten the development of therapeutics presenting improved efficacy and increased selectivity against neurodegeneration, and improve outcomes in patients with age-related cognitive aging and neurodegenerative disease conditions like AD.

## Figures and Tables

**Table 1:** Shared molecular markers of aging- and Alzheimer’s disease (AD)-associated microglial states. Selected genes reported to be altered in microglial populations during physiological aging and in AD or related neurodegenerative conditions, together with their principal functions. These markers are enriched in specific reactive microglial subsets and are not necessarily uniformly expressed across all microglia or specific to a single microglial state. APOE, apolipoprotein E; TREM2, triggering receptor expressed on myeloid cells 2; TYROBP, transmembrane immune signaling adaptor/DAP12; CLEC7A, C-type lectin domain-containing 7A; CST7, cystatin F; ITGAX, integrin subunit alpha X/CD11c; AD, Alzheimer’s disease.

Gene	Gene Function	References	
		Aging	Alzheimer’s Disease
*APOE*	*APOE* encodes a lipid-transport protein that regulates cholesterol in the brain	Mouse model [168]	Human [169,175]
*TREM2*	*TREM2* is a microglial receptor that is suggested to detect lipids, apoptotic-cell debris, and other damage-associated signals.	Mouse model [174]	Human and mouse study [176] Primarily mouse study, with supporting observations involving human TREM2 haploinsufficiency [170]
*TYROBP/DAP12*	*TYROBP (DAP12)* is an intracellular signaling adaptor used by TREM2 and other myeloid receptors.	Mouse model [174]	Human [177] Mouse model [178]
*CLEC7A*	*CLEC7A* is a pattern-recognition receptor expressed by myeloid cells.	Mouse model [168]	Mouse model [178]
*CST7*	*CST7* encodes cystatin F, a lysosomal protease inhibitor that helps regulate proteolytic activity in immune cells.	Mouse model [168]	Human and mouse model [178]
*ITGAX (CD11c)*	*ITGAX* encodes the CD11c integrin subunit, which contributes to cell adhesion, migration, complement recognition, and phagocytosis	Mouse model [168,174]	Human [178] Mouse model [178]

## Data Availability

No new data were created or analyzed in this study.

## Abbreviations

AD	Alzheimer’s disease
ADAPT	Alzheimer’s Disease Anti-Inflammatory Prevention Trial
APOE	apolipoprotein E 4
ARIA	Amyloid-Related Imaging Abnormalities
ASC	apoptosis-associated speck-like protein containing caspase recruitment domains
Aβ	amyloid-beta
BBB	blood–brain barrier
C1q	complement component 1q
CB	cannabinoid receptor
CCL3/4	macrophage inflammatory protein1α/β
CD33	cluster of differentiation 33
CD68	cluster of differentiation 68
CD83	cluster of differentiation 83
ChIP-Seq	chromatic immunoprecipitation sequencing
CNS	central nervous system
CRISPR	clustered regularly interspaced short palindromic repeats
CSF	cerebrospinal fluid
CSP	canonical senescence pathway
CST7	cystatin F
CX3CR1	cell surface chemokine receptor 1
CyTOF	comprehensive multiplexed mass cytometry
DAM	disease-associated microglia
DNA	deoxyribonucleic acid
ER	endoplasmic reticulum
GWAS	genome-wide association studies
HIF1α	mTOR-hypoxia-inducible factor-1α
IBA1	ionized calcium-binding adapter molecule 1
IFI16	interferon gamma inducible protein 16
IMG	human hPSC-derived microglia
IRF8	interferon regulatory factor 8
ITGAX; CD11c	integrin subunit alpha X
JAK	Janus kinases
LDAM	Lipid droplet accumulation microglia
LPL	lipoprotein lipase
mAbs	monoclonal antibodies
MCI	mild cognitive impairment
MGnD	microglial neurodegenerative phenotype
MHC	major histocompatibility complex
miRNA, miR	microRNAs
MMSE	Mini-Mental State Examination
MRI	magnetic resonance imaging
MRs	master transcriptional regulators
mTOR	mechanistic target of rapamycin
NFKB1	nuclear factor kappa b subunit 1
NFTs	neurofibrillary tangles
NK	natural killer
NLRP3	NOD-like receptor family, pyrin domain-containing 3
NSAIDS	non-steroidal anti-inflammatory drugs
OPCs	oligodendrocyte progenitor cells
p16	cyclin-dependent kinase inhibitor 2A/p16INK4A
P22	subtype-selective sialic acid mimetic
P2RY12	purinergic receptor P2Y, G-protein coupled, 12
P2X7R	purinergic P2X7 receptor
p38 MAPK	p38 mitogen activated protein kinase
PD-1	programmed cell death protein 1
PET	positron emission tomography
PLIN2	lipid droplet surface protein perilipin 2
PPARy	peroxisome proliferator-activated receptor gamma
PS19	mouse tauopathy model expressing MAPT P301S
PU.1	purine rich box binding protein 1
RONS	reactive oxygen and nitrogen species
ROS	reactive oxygen species
RUNX1	runt-related transcription factor 1
SASP	senescence-associated secretory phenotype
SA-β-gal	senescence-associated β-galactosidase
scRNA-seq	single-cell RNA sequencing
SIRT1	sirtuin 1
snRNA-seq	single-nucleus RNA sequencing
STAT	signal transducer and activator of transcription proteins
sTNF	soluble TNF
TAL1	T-cell acute lymphocytic leukemia protein 1
TGFB	transforming growth factor-β
TMEM119	transmembrane protein 119
TNF	tumor necrosis factor
TREM2	Triggering receptor expressed on myeloid cells 2
TREM2+/−	TREM2 haplodeficiency
TYROBP	TYRO protein tyrosine kinase binding protein
WHO	World Health Organization
